# Histopathological correlations to ureteral lesions visualized during ureteroscopy

**DOI:** 10.1007/s00345-017-2035-3

**Published:** 2017-04-12

**Authors:** Søren Kissow Lildal, Flemming Brandt Sørensen, Kim Hovgaard Andreassen, Frederikke Eichner Christiansen, Helene Jung, Malene Roland Pedersen, Palle Jörn Sloth Osther

**Affiliations:** 1 Department of Urology, Urological Research Centre, Lillebaelt Hospital, Kabbeltoft 25, 7100 Vejle, Denmark; 2 Department of Clinical Pathology, Lillebaelt Hospital, Vejle, Denmark; 3 Institute of Regional Health Research, University of Southern Denmark, Vejle, Denmark

**Keywords:** Complications, Histopathology, Post-ureteroscopic lesion scale, Ureteral access sheath, Ureteral lesions, Ureteroscopy

## Abstract

**Purpose:**

To correlate ureteral lesions visualized during ureteroscopy with histopathological findings.

**Materials and methods:**

Ureteral access sheaths (UAS) sized 13/15 Fr. were inserted bilaterally in 22 laboratory pigs. During retraction of the UAS with a semirigid ureteroscope inside, ureteral lesions were evaluated and registered using the Post-ureteroscopic lesion scale (PULS). Ureters were excised in vivo between the uretero–pelvic junction and the uretero–vesical junction. Embedded in paraffin, 4-µm thick sections were step sectioned at 250–300 µm intervals and haematoxylin and eosin (HE) stained. Histopathological scoring of ureteral wall lesions was subsequently performed according to PULS.

**Results:**

In 72.1% of ureters, the highest histopathological score was at least 1 grade higher than the highest endoscopic PULS score. For 12 (27.9%) lesions, the difference was 2 scores higher, and for 1 (2.3%), it was 3 scores higher. The histopathological PULS grade was higher than the endoscopical PULS grade at all minimum, quartile, and maximum scores. There was a significant difference in the distribution of highest lesional scores between the endoscopic and histopathological PULS (*p* = 0.002). The calculated mean of the highest scores was 1.49 for endoscopic PULS and 2.51 for histopathological PULS (*p* < 0.0001).

**Conclusion:**

Histopathological evaluation of ureteral wall lesions after UAS placement revealed a significantly higher degree of severity than observed endoscopically. Thus, endoscopy underestimated the histopathological extent of the lesion in the majority of cases.

## Introduction

Retrograde intrarenal surgery (RIRS) is widely used for treatment of kidney stones and for diagnosis and treatment of other intrarenal pathologies. The endoscopes and ureteral access sheaths (UAS) that are used for the procedures are continuously getting smaller, but the risk of harming the ureteral wall when inserting an instrument to access the renal pelvis persists [1, 2]. Animal studies have shown that ureteral dilation with endoscopes and UAS as well as by ureteral obstruction can decrease perioperative ureteral blood flow, and subsequently cause inflammation, necrosis, and ureteral wall thickening with collagen deposition [3–5].

Recently, prospective clinical studies have focused on the risk of having a ureteral lesion from retrograde procedures done with the aid of UAS. In a series of 359 patients, UAS sized 12/14 Fr. caused ureteral injury in 46.5%, of which 13.3% involved the ureteral muscular coat [1]. A 101 patient series on UAS sizes 9.5/11.5 and 12/14 Fr. found mucosal damage in 38.6% and smooth muscle layer lesions in 2.9% [2]. In a study of 148 preoperatively JJ-stented cases using 14–16 Fr. UAS, 39.9% had superficial mucosal lesions, 17.6% had deeper mucosal lesions, and 4.7% patients had circumferential perforation [6]. These reports show that ureteral damage does occur. In order to perform uniform clinical decisions, grading systems for objective evaluation of postoperative ureteral lesions have recently been proposed [1, 7]. For this purpose the Post-ureteroscopic lesion scale (PULS) was developed [7]. PULS is a validated system with high inter-rater reliability for endoscopically assessable ureteral lesions. Although the system, if used correctly, offers a clinical tool that directs the attention of the surgeon to prevent possible postoperative complications, we hypothesized that direct observation of visible lesions may in some cases only be a sign of greater underlying damage.

The aim of this study was to correlate endoscopically evaluable ureteral lesions to the corresponding histopathological lesion in order to further validate ureteral lesion grading systems and improve the quality of clinical decisions.

## Materials and methods

### Experimental animals

The animal protocols were approved by The National Animal Experiments Inspectorate (Copenhagen, Denmark). Studies were performed on 22 anesthetized female pigs weighing 55 kg (Påskehøjgård, Ølsted, Denmark). The pigs were fed a standard diet during breeding. Before the study, they had access to water but were fasting 12 h prior to anesthesia.

After premedication with azaperone (4 mg/kg) and midazolam (4 mg/kg), anesthesia was induced by propofol (4–20 mg/kg) and maintained with sevoflurane (1.2 MAC) and fentanil (0.03 mg/kg/h). The pigs were orotracheally intubated and mechanically ventilated (GE Healthcare S5 Avance). Hydration was maintained by administration of saline (9 g/l sodium chloride; 10 ml/kg/h) at a temperature of 37 °C through an ear vein.

**Fig. 1:**
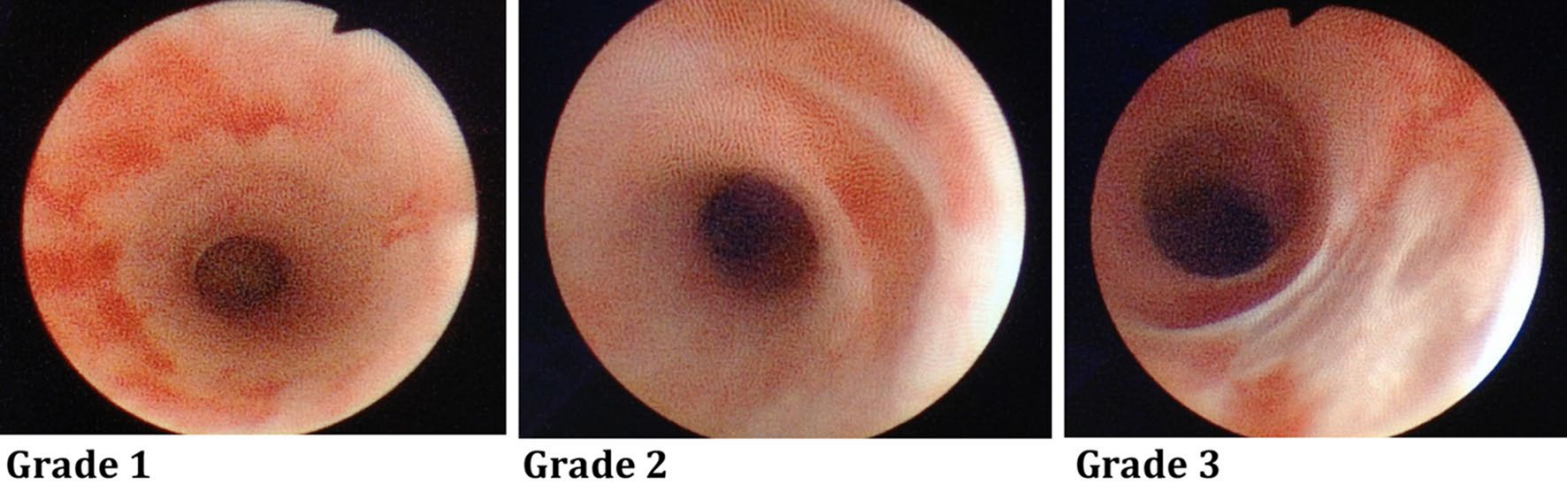
Examples of Endoscopic PULS grade 1, 2, and 3 lesions as visualized during endoscopic video recordings (see Table 1)

A cystoscope was inserted through the urethra into the bladder. A ureteral catheter (Selectip^®^, Bard Medical, Covington, Georgia, USA) was placed in the distal part of the ureter on one side, and a retrograde pyelography was performed to visualize the anatomy of the upper urinary tract. A guidewire (0.035 inches/150 cm, Sensor^®^, Boston Scientific, Marlborough, MA, USA) was inserted via the ureteral catheter to the renal pelvis, and the cystoscope was removed. The ureter size of the type of pigs evaluated in the present study was found to be slightly larger than the normal human ureter. In a pilot series, the clinical feeling of placing a 13/15 Fr. UAS in the pig ureters was equivalent to placing 12/14 Fr. UASs in human ureters, and therefore this UAS size was chosen as the appropriate size for the experiment. Over the guidewire, under fluoroscopic guidance, a hydrophilic UAS (13/15 Fr × 36 cm, Navigator™ Ureteral Access Sheath, Boston Scientific, Marlborough, MA, USA) was inserted. During retraction of the UAS with a semirigid ureteroscope (Karl Storz Endoskope, Tuttlingen, Germany, 7 Fr.) inside, ureteral lesions were evaluated and graded according to the PULS classification system (endoscopic PULS) [7] (Table 1; Fig. 1). The insertion and extraction procedure was performed bilaterally. All procedures were performed by two experienced endourologists (KHA, PJSO).

**Table 1:** Endoscopical and histopathological scoring of ureteral lesions based on the Post-Ureteroscopic Lesion Scale (PULS) (10) with a few modifications (see text)

PULS grade	Endoscopic PULS	Histopathological PULS
Grade 0	No lesion	No mucosal lesion seen. However, minor mucosal edema, luminal fibrin casts and/or bleeding, and/or mucosal molding, without any architectural mucosal damage or abrasions may be seen
Grade 1	Superficial mucosal lesion and/or significant mucosal edema/hematoma	Superficial mucosal damage mandatory, typically in the form of minor mucosal tears with significant edema and/or bleeding
Grade 2	Sub-mucosal lesion	Mucosal and sub-mucosal lesions, often with deep tears, however, with no involvement of the muscular coat of the ureter
Grade 3	Perforation with less than 50% partial transection	Deep localized penetrating tears, breaking through the muscular coat of the ureter, but involving less than 50% of the ureteral circumference.
Grade 4	More than 50% partial transection	Like Grade 3, but involving more than 50% of the ureteral circumference
Grade 5	Complete transection	Complete transection of the ureter

Through a midline abdominal incision, both ureters were exposed and carefully excised in vivo including surrounding connective tissue between the uretero–pelvic junction and the uretero–vesical junction.

Each ureter was immersed and stored in neutral, buffered formalin 10% (Hounisen, DK) for preservation until histopathological processing in the laboratory.

Finally, the pigs were euthanized under anesthesia with 20 ml of pentobarbital, 200 mg/ml.

### Tissue processing

**Fig. 2:**
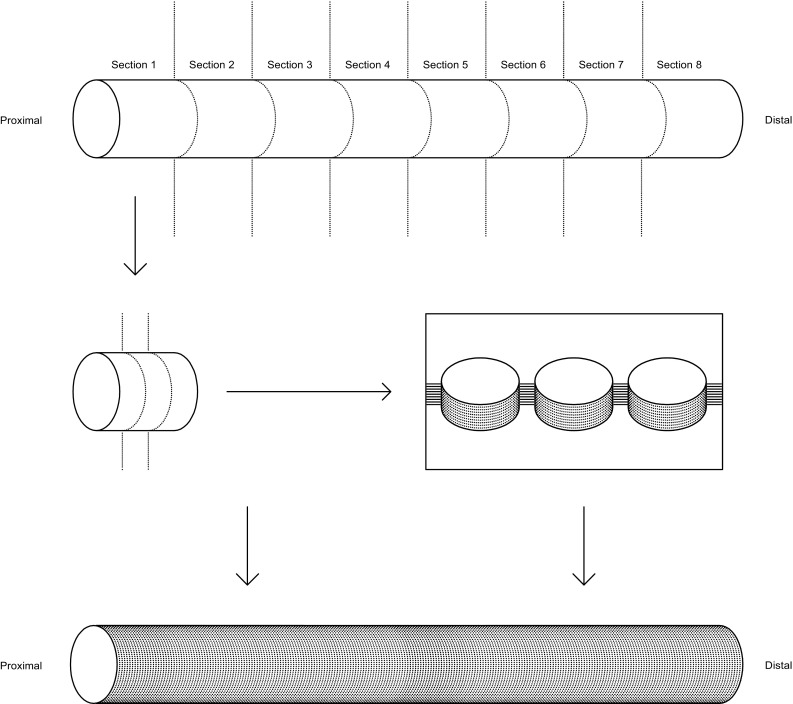
After fixation, the ureters were cut at right angle to the tubular organ into pieces of equal length. Each of these pieces were then further cut into 3 pieces, and each triplet was placed in one tissue container, routinely processed, and embedded in paraffin. Four-µm thick sections were step sectioned at 250–300 µm intervals, producing step sections from ≥10 levels. Thus 240 histologic sections (120–210 for the 6 shorter ureters) were available from each ureter, covering the whole length of the organ

After fixation, the ureters were cut at right angle to the tubular organ into pieces of equal length. 37 of the ureters were cut into 8 pieces, but 6 of the harvested ureters were shorter in length, thus yielding only 7, 6, and 4 pieces of equal length. Each of these pieces were then further cut into 3 pieces, and each triplet was placed in one tissue container, routinely processed, and embedded in the same paraffin (Tissue-Tek Paraform™, Sakura, DK) block. Four µm thick sections were step sectioned at 250–300 µm intervals, using a rotation microtome, producing step sections from ≥10 levels, which were mounted on Superfrost Plus™ slides (Menzel-Gläser, Thermo Scientific, DK). After rehydration, the histological sections underwent staining with Mayer’s haematoxylin and eosin (HE), followed by dehydration and were cover slipped, using Pertex™ (Sakura, DK). Thus 240 histologic sections (120–210 for the 6 shorter ureters) were available from each ureter, covering the whole length of the organ (Fig. 2).

### Histopathological scoring of ureteral lesions

**Fig. 3:**
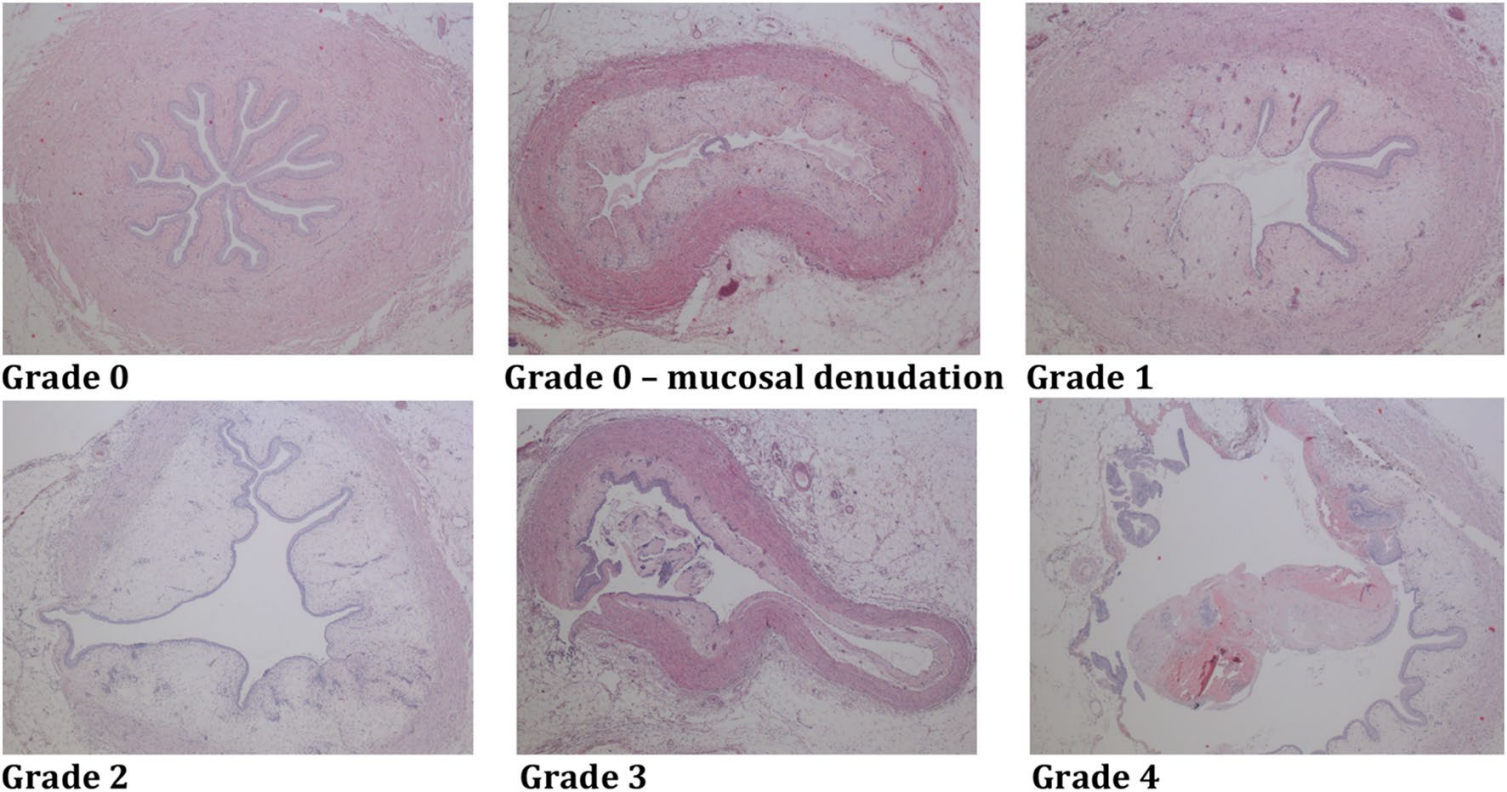
Examples of histopathological PULS grade 0–4 lesions (see Table 1)

The pathologist was blinded to the endoscopic PULS grade. Scoring of histopathological ureteral lesions was based on PULS (histopathological PULS) [7] (Table 1; Fig. 3), with a few modifications, as summarized in Table 1. In the case of tangential sections with no possibility to evaluate the whole ureteral circumference, a score of “not determined” (ND) was given, unless a significant lesion (histopathological PULS ≥1) was seen in the remaining part of the evaluable ureteral wall.

The highest endoscopic PULS and the highest histopatholocial PULS for each ureter were deducted as the primary outcome measures in order to compare the results of the two scoring modalities.

### Statistical analysis

Statistical analysis was performed with Stata (StataCorp, Texas) comparing mean and quartile scores, and using Wilcoxon signed-rank test for significance. Fisher’s exact test was used for analysis of difference in the distribution of highest lesional scores; *p* < 0.05 was considered significant.

## Results

A total of 44 ureters were harvested from the experimental animals. Due to a technical error, no intraoperative evaluation was obtained from one of the ureters and this specimen was excluded, yielding 43 samples for final analysis. A total of 3310 histological sections were morphologically evaluated. 65 (2%) of the sections were cut tangentially, making them incomplete in ureteral circumference, and therefore not determinable (ND) regarding lesional score (Table 2).

**Table 2:**
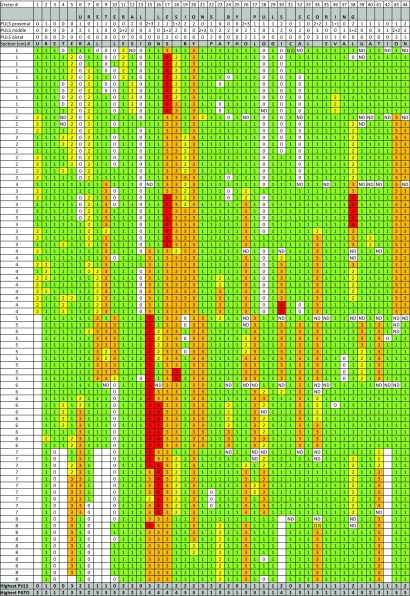
Complete overview of all registered lesional scores by endoscopic PULS and histopathological evaluation

Highest lesional score for each ureter is shown in the *bottom two lines*. Histopathological scores are arranged in columns and color coded (*1* = *green*, *2* = *yellow*, *3* = *orange* and *4* = *red*red), creating a numerical visual image of each ureter. Ureter #14 was excluded from analysis due to missing intraoperative endoscopic evaluation. *Six* of the ureters were shorter in length than the rest (*empty boxes*)

All endoscopically visible lesions were scored using the PULS grading system and were assigned an endoscopic PULS score [2], and it was registered using endoscopy and fluoroscopy whether the lesion was found in proximal, middle, or distal ureter. Histopathological PULS scoring for the whole ureter in each individual ureteral unit is presented in Table 2, creating a numerical visual image of the lesions.

Endoscopic PULS ranged between 0 and 3 compared to histopathological PULS ranging between 0 and 4. In 8 of 43 ureteral units, no lesions were observed endoscopically (18%). Histopathologically, no ureteral units were without lesions.

Comparing the highest endoscopic PULS score with the highest histopathological PULS score, only in 1 (2.3%) of the ureters, the endoscopic PULS score was 1 point higher than the histopathological score, and in 11 ureters (25.6%), they were equal (Table 2). In the remaining 31 ureters (72.1%), the histopathological PULS was at least 1 grade higher than the endoscopic PULS score. For 12 (27.9%) of these, the difference was 2 score points, and for 1 (2.3%), it was 3 points.

Statistical analysis using only the highest lesional scores showed that there was a highly significant difference between endoscopic and histopathological PULS grading (*p* = 0.002) (Table 3). The histopathological PULS score was at least 1 grade higher than the endoscopic PULS grade for minimum, quartile, and maximum scores (Table 3). The calculated mean of the highest scores was 1.49 for endoscopic PULS and 2.51 for histopathological PULS score (*p* < 0.0001).

**Table 3:** Mean, minimum, quartile, and maximum scores for Endoscopic PULS (Endo PULS) and histopathological PULS (Histo PULS)

	N	Mean	Min	25% percentile	50% percentile	75% percentile	Max
Endo PULS	43	1.49	0	1	2	2	3
Histo PULS	43	2.51	1	2	3	3	4

In order not to restrict analysis to the highest registered lesional score for each ureter, we also calculated the mean lesional score for each ureter, thereby comparing the longitudinal extent of lesions registered by the two evaluation systems. For the histopathological PULS scoring, mean scores were calculated as simple means of all evaluation scores. For the endoscopic PULS scores, we used video recordings during extraction of the UAS and ureteroscope to estimate the length of all registered lesions. The length was recorded as being <10%, <20%, <30%, or <40% of the total length of the ureter. If estimation of the length was difficult or uncertain between two intervals, the higher of the two intervals was chosen as the registered length in order not to underestimate the lesion length. The registered length of all lesions was used to calculate a weighted average lesional score for each ureter regarding endoscopic PULS. Finally, a total material, mean lesional score was calculated (Table 4). In all 43 ureteral units, the mean lesional score was higher by histopathological evaluation. The whole material mean endoscopic PULS score was 0.5, and the whole material mean histopathological PULS score was 1.4 (*p* < 0.001).

**Table 4:** Mean endoscopic (Endo PULS) and histopathological (Histo PULS) PULS for each ureter, and mean scores of Endo PULS and Histo PULS evaluations of the whole material

Ureter #	1	2	3	4	5	6	7	8	9	10	11	12	13	15	16	17	18	19	20	21	22	23
Mean Endo PULS	0.0	0.1	0.0	0.0	0.3	0.3	0.2	0.2	0.1	0.0	0.6	0.4	0.0	1.4	0.6	1.2	0.9	0.4	0.6	0.5	0.5	0.2
Mean Histo PULS	1.5	1.1	0.8	1.1	1.7	1.1	1.1	1.4	1.8	0.7	1.1	1.1	0.5	2.6	1.9	3.2	2.5	1.5	2.9	1.5	1.0	1.2

Ureter #	24	25	26	27	28	29	30	31	32	33	34	35	36	37	38	39	40	41	42	43	44	Whole material mean
Mean Endo PULS	0.2	0.0	1.6	0.4	0.1	0.2	0.6	0.0	0.5	0.0	1.6	0.4	0.6	0.2	0.8	0.2	0.2	0.7	0.2	1.5	0.9	0.5
Mean Histo PULS	1.1	1.0	2.1	1.5	0.8	1.0	1.3	1.0	1.3	1.0	2.0	1.0	1.0	1.0	1.7	1.2	1.0	1.7	1.0	1.8	1.7	1.4

The PULS grading system by default only applies to endoscopically assessable injuries. To take this into account, we performed a downgrading of the highest histopathological PULS score from 1 to 0 in 5 cases where the highest endoscopic PULS score was 0. In all cases where the highest histopathological lesional score was >2, no changes were made as lesions of this severity by definition should be endoscopically assessable. By performing this adjustment, the number of ureters, in which the highest endoscopic PULS and histopathological PULS scores were equal, changed from 11 (25.6%) to 16 (37.2%), and the number of ureters, in which the histopathological score was at least 1 grade higher than the endoscopic PULS score, changed from 31 (72.1%) to 26 (60.5%). With this modification, the difference between the highest score between endoscopic and histopathological PULS was still highly significant (*p* < 0.001).

## Discussion

This porcine model attempted to simulate the conditions that exist during clinical RIRS with UAS usage. The study was performed in vivo on upper urinary tracts of pigs because of the anatomical and physiological similarities to conditions in humans, and the animal size was chosen to approximate the size of the ureter of an adult human. All procedures were performed meticulously with respect for ureteral integrity as if they were done in a clinical setting.

To our knowledge, this is the first study reporting on histopathological correlations to endoscopically visualized ureteral lesions. The main finding of our study was that endoscopic grading of ureteral lesions using PULS following UAS usage underestimated the actual histopathological lesions. The exact clinical implication of this finding is uncertain. It may explain the clinical observation that some patients surprisingly develop a rigid, fibrotic ureter after previous retrograde endoscopic procedures, although no obvious lesions were encountered during the initial procedures. Long-term data of patients, primarily endoscopic PULS graded, are warranted for further evaluation of the clinical importance of the present data.

The endoscopic PULS scoring revealed severe lesions (grade 3) in 7 of the ureteral units (16.2%), which is comparable to results reported in clinical studies using UAS sized 12/14 Fr. [7, 1], confirming that our animal model with usage of 13/15 Fr. UASs was comparable to the human clinical setting.

In the histopathological evaluation process, the differentiation between PULS scores 3 and 4 sometimes proved to be challenging, as the degree of circumferential involvement was difficult to estimate. The same applied to the differentiation between scores 2 and 3, where a distinction should be made based upon the depth of the lesion, which was especially difficult in cases where the ureter was very dilated. In all cases where the exact histopathological lesional grade was questionable, we chose to downgrade the severity. In Table 2, which simulates an “image” of the ureter in terms of histopathological lesions, it is noticeable that the longitudinal distribution of lesions was quite coherent and not interrupted by observations of markedly different severity, which corresponds with the clinical feeling of a smooth passage of the UAS without sensation of resistance. We did however at times notice abrupt transitions between grade 1 and 3, where a gradual transition would have been expected (Table 2: ureteral units 9, 32, and 43). This may have occurred due to forced introduction of the UAS during a ureteral contraction (peristalsis), which was not noticed by the surgeon. It is well known that rising pressure in the renal pelvis may promote peristalsis due to activation of pacemaker cells [8]. Overfilling of the collecting system thus may be involved in access-related ureteral injuries [8].

During histopathological scoring of lesions, other observations were made, such as profound ureteral dilatation sometimes accompanied by total epithelial denudation but without lesion of the muscular coat; de-epithelialization with luminal fibrin casts; sub-mucosal edema and hematoma; acute neutrophil inflammation often with angiocentric location; and focal necrosis. These findings correspond with previous findings demonstrating a marked up-regulation of proinflammatory mediators in the ureteral wall following UAS usage [5]. Interestingly, we also saw varying degrees of focal eosinophilia, at times very profound and located under the mucosal or deep in the muscular layers. We do not know of any reason why the laboratory animal should present with eosinophilia in the ureter, but we did sometimes observe luminal foreign bodies of gel-like appearance, which may be remnants of the hydrophilic coating the UAS. Whether this may have any implications for postoperative outcome needs to be further evaluated.

Post-ureteroscopic ureteral lesional scores have in part been developed as an instrument to decide whether the patient should be stented or not [1, 2, 7]. In 19 (44.2%) of cases in our study, the PULS grade was upgraded from superficial to severe (from grade 1 or 2 to grade 3 or 4) by histopathological evaluation. This suggests that some of the observed superficial lesions could possibly represent penetrating lesions and as such accordingly should be followed by JJ-stenting. Whether our observations also may have clinical implications for immediate as well as long-term outcome with regard to ureteral function needs to be evaluated in a clinical setting using ureteral lesional scoring.

## Conclusions

Endoscopical evaluation of ureteral lesions using the PULS grading system following UAS placement in a porcine model underestimated the actual histopathological lesion. The findings may explain why ureteral malfunction occurs in some individuals following ureteral instrumentation.
